# Quantitative Assessment of Peripheral Nerve Echogenicity in Children and Adolescents Aged 2–17 Years: A Retrospective Cross-Sectional Ultrasound Study

**DOI:** 10.3390/jcm15083051

**Published:** 2026-04-16

**Authors:** Jan-Hendrik Stahl, Charlotte Schubert, Anna-Sophie Grimm, Lina Maria Serna-Higuita, Cornelius Kronlage, Julia Wittlinger, Magdalena Schühle, Natalie Winter, Alexander Grimm

**Affiliations:** 1 Department of Neurology and Epileptology, Hertie Institute for Clinical Brain Research, University of Tübingen, 72076 Tübingen, Germany; 2 Department of Pediatric Neurology, University Children’s Hospital Tübingen, 72076 Tübingen, Germany; 3 Department of Clinical Epidemiology and Applied Biostatistics, University of Tübingen, 72076 Tübingen, Germany

**Keywords:** grayscale, reference values, image processing, computer assisted, diagnostic imaging, peripheral nervous system diseases

## Abstract

**Introduction/Aims**: Quantitative analysis of nerve echogenicity can support the diagnosis of mono- and polyneuropathies, for instance by distinguishing inflammatory-demyelinating from axonal damage. However, echogenicity is mainly assessed qualitatively and examiner-dependently. This study aimed to establish quantitative reference data for grayscale values of peripheral nerves in the upper and lower extremities of healthy children and adolescents to provide a clinical benchmark. **Methods**: We retrospectively analyzed ultrasound data from 211 healthy children aged two to seventeen years who had undergone standardized examinations of 15 peripheral nerve sites. Grayscale analysis (0–255 levels per pixel) was performed within manually defined regions of interest (ROIs) using ImageJ (version 1.52). Echogenicity values were correlated with age, weight, height, and body mass index (BMI). **Results**: Echogenicity showed no significant overall association with biometric parameters. Mean grayscale values ranged from 85.23 ± 2.16 for the tibial nerve at the medial malleolus to 134.62 ± 2.69 for the sural nerve. Gain settings below 60 resulted in significantly lower grayscale values, whereas measurements with gain ≥ 60 were stable and comparable. **Discussion**: We propose reference grayscale ranges for peripheral nerves in healthy children and adolescents as a practical benchmark for clinical use and future studies. Due to technical constraints—particularly retrospective image processing and non-lossless data export—each laboratory should establish its own reference dataset, or multicentric parameters should be established. As our sample consisted predominantly of Caucasian participants, ethnic differences should be considered when applying these values to other populations.

## 1. Introduction

High-resolution ultrasound (HRUS) has become an established and guideline-recommended imaging modality in the evaluation of peripheral nerve disorders, for example, in the EAN/PNS guidelines on chronic inflammatory demyelinating polyradiculoneuropathy (CIDP) [1]. Among the various sonographic parameters employed, nerve cross-sectional area (CSA) has proven to be a highly reproducible parameter that reflects nerve enlargement and correlates with disease activity in CIDP [2,3,4,5,6,7,8]. However, nerve swelling is not a pathognomonic finding and can also be seen in several other neuropathies; among these, for instance, is Friedreich’s ataxia [9]. Inverse correlations are also possible, as has been demonstrated, for example, in individuals with Parkinson’s disease and parasympathetic dysfunction, where the CSA of the vagus nerve is associated with smaller nerves [10]. Consequently, CSA measurement has become a cornerstone in the diagnostic work-up of both mono- and polyneuropathies. HRUS assists in differentiating axonal from demyelinating neuropathies and inflammatory from non-inflammatory neuropathies [11] and provides valuable guidance in entrapment syndromes and traumatic nerve injuries, including pre- and postoperative assessment [12]. Reference values for nerve CSA at multiple anatomical sites have been established and reproduced in different ethnic groups [3,13,14,15,16].

Beyond nerve size, HRUS allows for detailed assessment of fascicular structure, epineural and perineural tissues, and intraneural vascularity using Doppler techniques [17,18]. More recently, shear-wave elastography (SWE) has expanded the diagnostic potential of nerve sonography by enabling quantitative evaluation of tissue stiffness [19,20].

In addition to these structural and mechanical parameters, nerve echogenicity has emerged as another promising sonographic marker. Alterations in echogenicity have been associated with various pathophysiological processes, including edema, demyelination, fibrosis, and axonal degeneration [5,21,22,23,24]. Qualitatively, hypoechoic nerves and fascicles have been described in inflammatory polyneuropathies and entrapment syndromes, whereas hyperechoic patterns are more commonly observed in chronic or fibrotic lesions, trauma, chronic, long-lasting CIDP variants, and some axonal neuropathies [23,25]. However, qualitative assessment remains subjective and depends on device presets, reference of tissue choice, and examiner experience [26].

To overcome these limitations, semi-quantitative methods for assessing nerve echogenicity have been proposed [21,27,28]. The largest available reference dataset to date includes five nerves in 79 healthy adults [29], but corresponding normative data for children and adolescents are lacking.

Therefore, the aim of the present study was to establish comparative values for quantitative peripheral nerve echogenicity of the upper and lower extremities in healthy children and adolescents aged 2 to 17 years. These data may provide a crucial reference for the clinical evaluation and longitudinal monitoring of pediatric patients with neuromuscular and peripheral nerve diseases.

## 2. Materials and Methods

We retrospectively analyzed data from our previously published studies on reference values for nerve ultrasound in children and adolescents [30,31]. In these studies, healthy participants aged between 2 and 17 years were recruited from local kindergartens and schools between May 2018 and May 2019. Written informed consent was obtained from parents or guardians, and the study protocol was approved by the local ethics committee on 1 March 2018 (Tübingen 765/2017BO). Individuals with developmental delays, known neurological disorders, or a positive family history of genetic polyneuropathy were excluded from the study, as were conditions that could promote the development of polyneuropathy (e.g., untreated or inadequately treated diabetes mellitus). Exclusion criteria were assessed using a medical questionnaire. High-resolution ultrasound (HRUS) examinations were originally performed by CS and ASG using a 14 MHz linear transducer (Mindray TE7, Ultrasound Systems, Darmstadt, Germany) following several months of dedicated training under the supervision of experts NW and AG. At the start of each examination, imaging parameters were optimized to ensure inter-subject comparability while maintaining optimal nerve visualization. The focus was aligned with the nerve and individually adjusted for each image. Apart from gain and focus, parameters such as depth, mechanical index, and dynamic range were kept constant for each participant. The detailed imaging protocol has been described previously [22,30,31].

In brief, the median and ulnar nerves were examined at the mid-forearm, elbow, and mid-upper arm, with the median nerve additionally evaluated at the wrist proximal to the carpal tunnel. The two terminal branches of the radial nerve were assessed around the elbow proximal to the arcade of Frohse. On the lower extremity, the tibial and fibular nerves were examined at the popliteal fossa and ankle joint. The cervical nerve roots C5 and C6 were evaluated longitudinally, lateral to the transverse processes, and the vagus nerve was examined within the carotid triangle. All examinations were performed on the participant’s right side.

**Figure 1:**
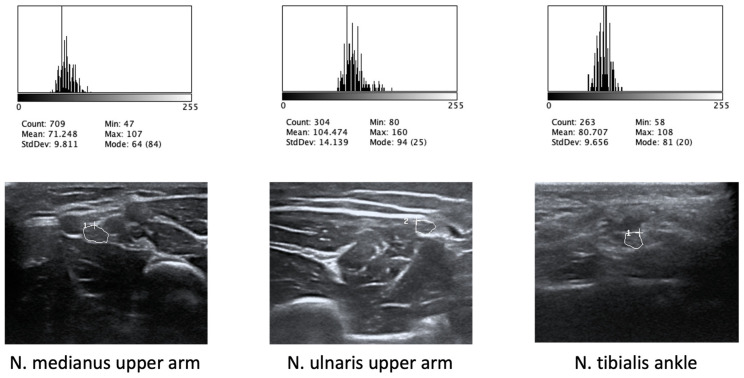
Sample ultrasound images with corresponding gray value histograms.

Given the known influence of insonation angle and probe pressure on echogenicity, care was taken to position the transducer perpendicular to the skin surface, applying only minimal pressure equivalent to the probe’s own weight. This was particularly challenging in regions where the nerve course was not straight, such as the tibial nerve at the ankle or the ulnar nerve at the elbow. An illustrative example of anisotropy is provided in the article by Ricci et al. on various ultrasound artifacts [32]. For our study, no specific aids were used to check that the ultrasound probe was positioned at a right angle. However, the examiners were informed in advance of the importance of this factor and trained accordingly. In addition, not all participants tolerated the entire ultrasound examination. For the present analysis, grayscale quantification was performed for the median (MN), ulnar (UN), radial (RN), tibial (TN), fibular (FN), sural (SN), and vagus (VN) nerves using public-domain image analysis software (ImageJ, version 1.52; National Institutes of Health, Bethesda, MD, USA;). Ultrasound images were exported from the scanner as JPEG files, and one non-blinded examiner (CS) manually delineated the region of interest (ROI) within the pre-circled cross-sectional area (CSA), including as much of the CSA as possible while excluding the bright measurement boundary line. To do so, the hypoechoic fascicles were manually outlined without including the hyperechoic epineurium, which is difficult to distinguish from the surrounding connective tissue. Pixel intensity values were converted to 8-bit grayscale (0 = darkest, most hypoechoic; 255 = brightest, most hyperechoic), and the mean grayscale value of each ROI was calculated. An illustrative example is provided in Figure 1. Images with labeling artifacts (e.g., white annotation letters overlapping the ROI) or with motion blur were excluded from the analysis. Echogenicity was determined directly from the ROI without normalization to surrounding tissue or background echogenicity.

The primary outcome of this study was to evaluate the effect of gain setting on nerve echogenicity. The secondary endpoint was to assess whether echogenicity was associated with demographic and anthropometric variables, particularly age and sex.

Demographic characteristics of the study cohort were summarized using descriptive statistics. Categorical variables were expressed as absolute and relative frequencies, while continuous variables were presented as means ± standard deviation (SD) with 95% confidence intervals or as medians and interquartile ranges (IQRs), as appropriate. Data normality was assessed using the Shapiro–Wilk test and visual inspection of histograms, boxplots, and P-P and Q-Q plots.

To evaluate the effect of gain settings, participants were assigned to three groups: group A (gain < 60), group B (gain 60–80), and group C (gain > 80). Univariate comparisons of grayscale values among the three groups were performed using one-way analysis of variance (ANOVA) for normally distributed data or the Kruskal–Wallis test for non-normal distributions. Pairwise post hoc comparisons were adjusted for multiple testing using the Bonferroni correction.

Associations between echogenicity and CSA, age, weight, height, and BMI were assessed using Pearson or Spearman correlation coefficients, as appropriate. Mean differences between echogenicity and age stratified into four groups (2–4, 5–7, 8–13, and 14–17 years) were further examined using one-way ANOVA. Residuals were inspected for normality by visual inspection of histograms and Q-Q plots. Homoscedasticity was evaluated using the Levene test; when residuals showed substantial deviation from normality—even after log transformation, or when homoscedasticity was not satisfied—nonparametric testing (Kruskal–Wallis) was applied.

All statistical analyses were performed using IBM SPSS Statistics version 25.0 (IBM Corp., Armonk, NY, USA) and R software version 3.6 (R Foundation for Statistical Computing, Vienna, Austria). Two-sided *p*-values ≤ 0.05 were considered statistically significant.

## 3. Results

Post-processing grayscale analysis of HRUS images was performed for a total of 233 children using ImageJ. Twenty-two subjects were excluded due to missing data in all measurements, leaving 211 children and adolescents for the final analysis. Nerves were evaluated at 15 anatomical sites. Descriptive statistics of the study population are summarized in Table 1. The number of examined subjects per nerve site ranged from 186 to 199, as not all participants tolerated the entire examination. Although ultrasound is painless and generally well accepted, it proved challenging for some of the youngest children despite the presence of a parent. A few additional images were excluded because labeling overlapped with the ROI.

**Figure 2:**
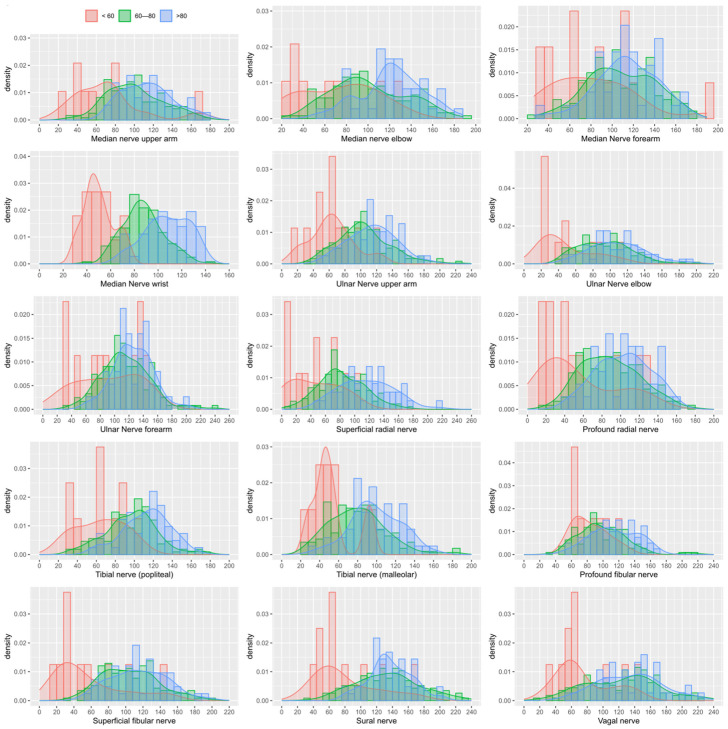
Distribution of grayscale values across different gain settings (groups A–C). Comparable distributions are observed for gains ≥ 60 (groups B, dyed green, and C, dyed blue), whereas markedly lower grayscale values occur when gains < 60 (group A, dyed red).

Statistically significant differences in mean grayscale values were mainly observed between group A (gain < 60) and group C (gain > 80) (Table 2 and Table 3). Histograms of grayscale value distributions by gain setting (Figure 2) were generated. These demonstrated high congruence between groups B (gain 60–80) and C (gain > 80), supporting the assumption that echogenicity remains stable within this gain range. In contrast, markedly lower grayscale values were observed in group A (gain < 60), indicating that insufficient gain leads to systematic underestimation of echogenicity. Contrary to the assumption that higher gain settings automatically increase grayscale intensity, Figure 2 shows comparable distributions for gains between 60 and 80 and >80.

Correlation analysis was evaluated by Pearson or Spearman correlation and revealed no significant associations between mean grayscale values and weight, except for the median nerve at the wrist, which showed a weak positive correlation (Pearson r = 0.237, 95% CI 0.103–0.363, *p* < 0.001). Similarly, age was not significantly correlated with echogenicity except for the median nerve at the wrist (Pearson r = 0.159, 95% CI: 0.024–0.288, *p* = 0.022) and the fibular nerve at the popliteal fossa (Pearson r = 0.149, 95% CI: 0.008–0.283, *p* = 0.038). Height correlated weakly only with the median nerve at the wrist (Pearson r = 0.158, 95% CI: 0.022–0.288, *p* = 0.023).

**Figure 3:**
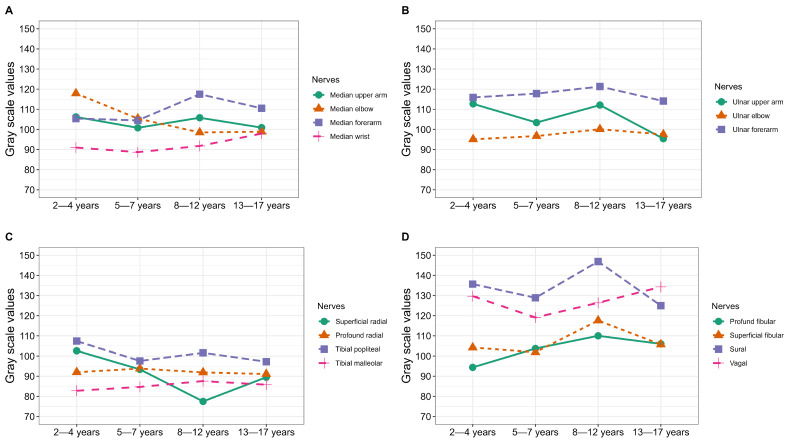
Grayscale values by age categories. (**A**) Mean gray-scale values of the median nerve at various sites on the upper and lower arm across different age categories. (**B**) Mean gray-scale values of the ulnar nerve at various sites on the upper and lower arm across different age categories. (**C**) Mean gray-scale values of the radial nerve at the elbow and of the tibial nerve on the leg across different age categories. (**D**) Mean gray-scale values of the fibular nerve at various sites on the leg, the sural nerve at the calf, and the vagal nerve on the neck, across different age categories.

Analysis of variance confirmed that age was not an independent predictor of mean grayscale values across any of the examined nerves (Table 4 and Table 5, Figure 3, Supplementary Table S1). As illustrated in Figure 3, no differences between echogenicity mean values and age stratified by group could be identified.

## 4. Discussion

In this retrospective study, we provide guiding data for peripheral nerve echogenicity in healthy children and adolescents aged 2 to 17 years. On an eight-bit grayscale (0–255), mean echogenicity values ranged approximately from 90 in larger nerves to 110–140 in smaller nerves. Our findings indicate that gain settings between 60 and >80, as well as demographic parameters such as age, sex, and BMI, do not meaningfully influence echogenicity measurements. Based on these results, we propose benchmark grayscale values for clinical orientation as a basis for future studies, using only images acquired with gain settings above 60. Even though the data presented suggest that there is a relevant change in echogenicity in the range of a gain < 60, this assumption still needs to be confirmed by further studies. The ultrasound images on which this study is based had already been processed by the ultrasound device software and were not designed to measure echogenicity. Consequently, other relevant parameters such as sound angle and depth were not standardized, which affects the delineation and, thus, the assessment of individual fascicles. This limits the actual clinical application of the values presented. Unlike the nerve cross-sectional area (CSA), which increases with age [30,31], nerve echogenicity appears to remain stable throughout childhood and adolescence. This behavior resembles that of muscle echogenicity, which has likewise been reported to show little change with growth and maturation [33]. While muscle echogenicity can be influenced by subcutaneous tissue thickness [34], no comparable association was found between nerve echogenicity and weight or BMI in our cohort, although this may in part reflect the small number of participants with elevated BMI. Nevertheless, Stolz et al. also concluded in their prospective observational study of 395 peripheral nerves in the upper extremities that BMI has no influence on echogenicity, although this study was based on a four-level categorization of echogenicity and no analysis of gray values was performed [27]. The statistically significant correlation between age and the echogenicity of the median nerve at the wrist and the fibular nerve at the popliteal fossa, as well as between body height and the echogenicity of the median nerve at the wrist, is likely to be of no clinical relevance.

Previous studies assessing nerve echogenicity often applied binary thresholding techniques, such as calculating the “fraction of black” [21,28,29]. In contrast, we quantified mean grayscale intensity to retain the full range of information in the images. Mean echogenicity values ranged from 85.2 ± 2.2 for the tibial nerve at the medial malleolus to 134.6 ± 2.7 for the sural nerve. Variability among nerves likely reflects both structural and technical factors: intrinsic differences such as connective tissue content, and external factors like adjustments in gain made by examiners to maintain consistent overall image brightness.

The resulting reference intervals, defined by 95% confidence limits, may help distinguish normal from hypo- and hyperechoic nerves at 15 predefined measurement sites in children. These data establish an important foundation for quantifying nerve echogenicity in pediatric neuromuscular disorders, particularly polyneuropathies, and for developing diagnostic thresholds in future research. Echogenicity analysis could enhance the diagnostic value of nerve ultrasound, complementing CSA measurements that are already widely used [30,31,35]. This may be especially beneficial in pediatric settings, where electrophysiological studies are often uncomfortable or difficult to perform [36].

A major limitation of this study is its retrospective and cross-sectional design. Since the evaluation is based on data from previous studies, it was not possible to perform a power analysis. In a prospective setting, ultrasound gain could also have been standardized to minimize variability. In addition, it would have been possible to measure the intra-individual development of echogenicity over time. Technical challenges further complicate echogenicity assessment in general, particularly tissue anisotropy: even slight deviations in probe angle can significantly affect echogenicity. The more organized a structure is, the greater its anisotropy. This phenomenon becomes evident when assessing the median nerve at the wrist. In this instance, minor adjustments in the transducer’s inclination result in substantial alterations to the echogenicity of the surrounding flexor tendons, surpassing the degree of change observed in the median nerve. Still, maintaining perpendicular probe orientation is especially difficult for curved or deep nerves, such as the tibial nerve at the medial malleolus or in the popliteal fossa. In their intermodal comparison of the representation of the fascicles of the median nerve, the ulnar nerve, and the superficial branch of the radial nerve in 14 body donors, Pusnik and colleagues demonstrated that HRUS occasionally lacks the capability to detect individual fascicles or erroneously represents nearby fascicles as a contiguous fascicle group. The resolution of HRUS is contingent upon various factors, including the frequency employed and the depth of the nerve to be imaged [37]. Consequently, it can be posited that gray value analysis is also influenced by the probe used, given the disparate imaging of intranerve structures and surrounding tissue. This limitation renders the general applicability of the proposed values somewhat restricted. Further, in our study, manually determining the ROI may also have had a slight influence on the echogenicity analysis. Moreover, the analysis was limited by the use of JPEG exports from the ultrasound system, resulting in some loss of image information due to non-lossless compression. Access to raw backscatter data would enable more precise and reproducible analysis [38], but it is not routinely available on most devices. In the context of calibrated backscatter analysis, the raw data undergoes analysis without the intervention of post-processing by ultrasound devices. The echogenicity values of a phantom, for example, can serve as a reference point, and various ultrasound devices can be calibrated to this phantom.

Recent methodological advances, such as calibrated muscle backscatter (cMB), have demonstrated that such quantitative grayscale analysis can be standardized across devices [38]. However, this approach requires knowledge of the decibel output and a calibration curve, which are not available for all ultrasound systems. The application of calibrated backscatter techniques may, therefore, represent a promising alternative for nerve assessment. Wu et al. demonstrated the high repeatability and reproducibility of this method in evaluating the median nerve at the wrist [39].

Another potential source of bias is the lack of a second examiner for echogenicity analysis, which would have been useful for assessing reproducibility, as well as the limited diversity of our study population, which primarily consisted of European children who grew up in southern Germany close to our university hospital. As ethnic differences in nerve morphology have been shown for CSA [40], similar effects on echogenicity cannot be excluded. Therefore, our guiding values should be interpreted cautiously when applied to non-European populations.

Despite these limitations, our data demonstrate that nerve echogenicity remains stable across a practical range of gain settings commonly used in clinical nerve ultrasound. Larger nerves tended to appear more hypoechoic, likely reflecting their thicker and more numerous fascicles. A comparative histopathological study could provide clarity on this hypothesis in the future. Although further refinement and standardization are desirable, echogenicity measurement in its current form is a feasible, straightforward, and potentially valuable extension of routine nerve ultrasound in children. Still, future prospective and longitudinal studies using fixed ultrasound protocols and comparing the gray values obtained from different ultrasound devices are necessary to verify the clinical relevance of quantitative echogenicity analysis. In addition, such studies should systematically assess intra- and inter-rater reliability of both image acquisition and post-processing procedures to ensure methodological robustness and reproducibility of quantitative echogenicity measurements. Subsequent to this, the applicability of echogenicity analysis for differentiating between healthy and diseased nerves must be demonstrated.

## Figures and Tables

**Table 1:** Baseline characteristics.

Variables	*n*	*n* (%)	
Sex	211		
female		104 (49.3)	
male		107 (50.7)	
Handedness	211		
right		184 (87.2)	
left		18 (8.5)	
uncertain		9 (4.3)	
Native language	211		
German		173 (83)	
Arabic		4 (1.9)	
polish		3 (1.4)	
Romanian		1 (0.5)	
Russian		2 (0.9)	
Turkish		1 (0.5)	
Uzbek		3 (1.4)	
bilingual (German/x)		24 (11.4)	
**Variables**	*n*	**Mean (±SD)**	**Min/Max**
Age (years)	211	8.18 (4.81)	2/17
Height (m)	210	1.31 (0.28)	0.80/1.91
Weight (kg)	204	31.92 (25.40)	10.50/111
BMI (kg/m^2^)	204	16.85 (3.10)	11.34/30.43

BMI = body mass index. “Uncertain” means that we did not receive a clear statement from the subject or the child’s parents. SD = standard deviation.

**Table 2:** Comparison of the gain groups of the individual nerve segments of the upper extremity.

Nerve Segment	Group	*n*	Mean	SD	Median	Group Comparison	Kruskal–Wallis Test	*p*-Value *
**Median nerve**								
upper arm	A	12	71.72	38.07	71.29	A vs. B	−2.945	0.058
	B	152	100.42	29.82	99.16	A vs. C	−4.151	<0.001
	C	46	113.95	26.18	11.90	B vs. C	−2.749	0.107
elbow	A	12	73.12	34.60	78.86	A vs. B	−2.194	0.509
	B	151	100.51	34.75	95.08	A vs. C	−3.959	<0.001
	C	45	121.94	29.24	120.30	B vs. C	−3.700	0.004
forearm	A	16	83.08	40.89	79.33	A vs. B	−2.685	0.392
	B	150	108.31	32.26	105.91	A vs. C	−3.056	0.121
	C	43	113.30	29.11	112.20	B vs. C	−1.091	0.999
wrist	A	14	50.13	12.73	47.61	A vs. B	−5.020	<0.001
	B	150	87.43	17.82	86.24	A vs. C	−7.609	<0.001
	C	45	107.57	18.55	108.39	B vs. C	−5.445	<0.001
**Ulnar nerve**								
upper arm	A	11	63.84	28.73	62.66	A vs. B	−3.471	0.028
	B	150	103.98	34.20	101.62	A vs. C	−4.282	<0.001
	C	49	114.03	30.03	113.61	B vs. C	−2.094	0.999
elbow	A	11	48.68	30.44	36.85	A vs. B	−3.750	0.003
	B	147	93.68	30.55	93.04	A vs. C	−4.829	<0.001
	C	50	108.18	31.92	106.48	B vs. C	−2.662	0.140
forearm	A	11	91.27	43.03	85.09	A vs. B	−1.473	0.999
	B	152	114.95	34.11	112.11	A vs. C	−2.505	0.221
	C	47	125.44	27.49	122.20	B vs. C	−2.271	0.417
**Radial nerve**								
deep	A	11	54.55	39.97	39.95	A vs. B	−2.688	0.129
	B	146	88.86	31.67	87.42	A vs. C	−3.789	0.003
	C	47	102.66	28.82	104.98	B vs. C	−2.556	0.191
superficial	A	11	46.13	33.97	48.13	A vs. B	−2.778	0.099
	B	146	82.62	31.98	78.72	A vs. C	−5.105	<0.001
	C	46	115.89	36.61	117.73	B vs. C	−4.997	<0.001
**Vagal nerve**								
	A	11	75.46	35.65	63.82	A vs. B	−3.224	0.023
	B	140	124.57	46.49	128.27	A vs. C	−3.718	0.004
	C	49	134.37	42.37	133.93	B vs. C	−1.422	0.999

SD = standard deviation. * *p* values adjusted using Bonferroni method.

**Table 3:** Comparison of the gain groups of the individual nerve segments of the lower extremity.

Nerve Segment	Group	*n*	Mean	SD	Median	Group Comparison	Kruskal–Wallis Test	*p*-Value *
**Tibial nerve**								
popliteal	A	10	70.20	28.64	70.10	A vs. B	−3.025	0.045
	B	135	97.33	27.40	100.47	A vs. C	−4.657	<0.001
	C	51	111.42	26.75	115.55	B vs. C	−3.767	0.003
malleolar	A	10	53.08	22.43	47.03	A vs. B	−2.648	0.146
	B	145	80.03	30.16	78.16	A vs. C	−4.460	<0.001
	C	53	99.08	26.59	95.63	B vs. C	−4.186	<0.001
**Fibular nerve**								
superficial	A	10	56.75	41.65	40.76	A vs. B	−3.294	0.018
	B	145	104.81	32.85	102.45	A vs. C	−4.044	<0.001
	C	52	114.58	32.29	109.81	B vs. C	−1.976	0.867
profound	A	9	83.68	21.88	78.46	A vs. B	−1.381	0.999
	B	136	99.65	31.24	96.02	A vs. C	−2.647	0.146
	C	51	112.88	30.19	111.29	B vs. C	−3.060	0.040
**Sural nerve**								
	A	10	80.97	40.68	65.18	A vs. B	−3.471	0.009
	B	145	133.63	40.99	131.64	A vs. C	−3.634	0.005
	C	52	137.33	27.00	133.92	B vs. C	−0.743	0.999

SD = standard deviation. * *p* values adjusted by Bonferroni.

**Table 4:** Grayscale values by age.

	Mean (±SD)				*p*-Value
	2–4 Years	5–7 Years	8–12 Years	13–17 Years	
**Median nerve**					
upper arm (*n* = 198)	106.2 (33.7)	100.8 (24.6)	105.8 (31.5)	100.9 (27.5)	0.681
elbow (*n* = 196)	117.9 (32.1)	105.4 (38.4)	98.5 (33.4)	98.9 (31.3)	0.017
forearm (*n* = 193)	105.4 (31.9)	104.5 (30.7)	117.5 (31.3)	110.5 (31.6)	0.131
Wrist (*n* = 195)	91.0 (18.1)	88.7 (22.6)	91.8 (16.5)	97.9 (21.5)	0.138
**Ulnar nerve**					
upper arm (*n* = 199)	112.7 (29.9)	103.4 (31.6)	112.1 (39.3)	95.4 (29.3)	0.040
elbow (*n* = 197)	95.1 (27.8)	96.7 (31.6)	100.1 (32.7)	97.5 (34.6)	0.881
forearm (*n* = 199)	115.9 (31.0)	117.8 (29.1)	121.3 (37.0)	114.1 (34.8)	0.777
**Radial nerve**					
superficial (*n* = 192)	102.6 (36.6)	93.4 (34.9)	77.5 (35.7)	89.5 (32.4)	0.006
deep (*n* = 193)	92.0 (30.1)	93.8 (33.1)	91.9 (29.9)	91.1 (34.1)	0.986
**Tibial nerve**					
popliteal (*n* = 186)	107.4 (25.8)	97.6 (24.6)	101.6 (28.9)	97.2 (32.8)	0.258
malleolar (*n* = 198)	82.8 (32.2)	84.7 (25.6)	87.6 (34.8)	85.8 (28.3)	0.884
**Fibular nerve**					
profound (*n* = 187)	94.4 (26.3)	103.7 (30.9)	110.0 (35.5)	106.0 (31.4)	0.100
superficial (*n* = 197)	104.2 (26.9)	101.9 (34.2)	117.6 (34.9)	105.7 (33.7)	0.068
**Sural nerve**					
(*n* = 197)	135.7 (30.8)	128.9 (38.1)	146.9 (42.2)	125.0 (36.1)	0.025
**Vagal nerve**					
(*n* = 189)	129.7 (41.5)	119.1 (46.5)	126.4 (50.9)	134.3 (41.8)	0.498

SD = standard deviation. All models were adjusted by gender.

**Table 5:** Upper and lower limits for grayscale analysis in nerves from healthy children.

	*n*	Mean	Lower CI 95%	Upper CI 95%	SD
**Median nerve**					
upper arm	198	103.57	99.43	107.70	2.10
elbow	196	105.43	100.54	110.32	2.48
forearm	193	109.42	104.94	113.91	2.27
wrist	195	92.08	89.27	94.88	1.42
**Ulnar nerve**					
upper arm	199	106.46	101.78	111.13	2.37
elbow	197	97.36	92.94	101.78	2.24
forearm	199	117.43	112.83	122.03	2.33
**Radial nerve**					
superficial branch	192	90.59	85.47	95.71	2.60
deep branch	193	92.22	87.75	96.69	2.27
**Tibial nerve**					
popliteal	186	101.27	97.23	105.30	2.04
malleolar	198	85.23	80.97	89.49	2.16
**Fibular nerve**					
profound branch	187	103.26	98.73	107.80	2.30
superficial branch	197	107.44	102.82	112.07	2.34
**Sural nerve**					
	197	134.62	129.32	139.92	2.69
**Vagal nerve**					
	189	127.11	120.57	133.64	3.31

CI = confidence interval.

## Data Availability

The original contributions presented in this study are included in the article/Supplementary Material. Further inquiries can be directed to the corresponding author(s).

## Supplementary Materials

The following supporting information can be downloaded at: https://www.mdpi.com/article/10.3390/jcm15083051/s1. Table S1: Analysis of Variance.

## Abbreviations

The following abbreviations are used in this manuscript:

ANOVA	one-way analysis of variance
BMI	body mass index
CI	confidence interval
cMB	calibrated muscle backscatter
CSA	cross-sectional area
HRUS	high-resolution ultrasound
IQR	interquartile range
MN	median nerve
P-P-plot	probability–probability plot
FN	fibular nerve
Q-Q-plot	quantile–quantile plot
RN	radial nerve
ROI	region of interest
SN	sural nerve
SWE	shear-wave elastography
TN	tibial nerve
UN	ulnar nerve
VN	vagal nerve
