# Supplementary material for: Quantitative Assessment of Peripheral Nerve Echogenicity in Children and Adolescents Aged 2–17 Years: A Retrospective Cross-Sectional Ultrasound Study
Source: J Clin Med. 2026 Apr 16;15(8):3051. doi: 10.3390/jcm15083051 (PMC13117568; doi:10.3390/jcm15083051)
Supplement: Supplementary file 1 [file jcm-15-03051-s001.zip › jcm-3986834-supplementary.pdf]

Supplemental Table 1 - Analysis of Variance

	β	95% CI	P values	Partial Eta^2
		Lower	Upper	
**Median Nerve**				
Upper arm				
Intercept	101.4			
5 – 7 years	-5.40	-19.21	8.40	0.008
8 – 12 years	-0.41	-14.21	13.40	
13 – 17 years	-5.30	-19.91	9.30	
Elbow				
Intercept	98.94			
5 – 7 years	-12.45	-28.47	3.57	0.052
8 – 12 years	-19.37	-35.31	-3.43	
13 – 17 years	-19.07	-35.92	-2.21	
Forearm				
Intercept	107.99			
5 – 7 years	-0.95	-15.77	13.86	0.029
8 – 12 years	12.05	-2.76	26.86	
13 – 17 years	5.06	-10.45	20.57	
Wrist				
Intercept	99.40			
5 – 7 years	-2.33	-11.56	6.90	0.028
8 – 12 years	0.76	-8.52	10.03	
13 – 17 years	6.85	-2.86	16.57	
**Ulnar nerve**				
Upper arm				
Intercept	96.996			0.042
5 – 7 years	-9.27	-24.62	6.09	
8 – 12 years	-0.61	-15.89	14.68	
13 – 17 years	-17.25	-33.50	-1.01	
Elbow				
Intercept	97.33			0.003
5 – 7 years	1.63	-13.20	16.45	
8 – 12 years	4.97	-9.79	19.72	
13 – 17 years	2.41	-13.37	18.19	
Forearm				
Intercept	115.7			0.006
5 – 7 years	1.93	-13.46	17.32	
8 – 12 years	5.40	-9.98	20.79	
13 – 17 years	-1.82	-17.99	14.35	
**Radial nerve**				
Superficial				
Intercept	88.72			0.065

5 – 7 years	-9.21	-25.95	7.52	
8 – 12 years	-25.10	-41.68	-8.53	
13 – 17 years	-13.12	-30.75	4.50	
Deep				
Intercept	94.68			0.001
5 – 7 years	1.82	-13.12	16.75	
8 – 12 years	-0.09	-14.88	14.70	
13 – 17 years	-0.86	-16.71	14.99	
**Tibial nerve**				
Popliteal				
Intercept	96.88			0.022
5 – 7 years	-9.81	-22.98	3.37	
8 – 12 years	-5.82	-19.06	7.42	
13 – 17 years	-10.15	-24.56	4.26	
Malleolar				
Intercept	86.07			0.003
5 – 7 years	1.84	-12.48	16.15	
8 – 12 years	4.79	-9.45	19.04	
13 – 17 years	3.00	-11.98	17.97	
**Fibular nerve**				
Deep				
Intercept	107.16			0.034
5 – 7 years	9.24	-5.51	23.98	
8 – 12 years	15.61	0.79	30.43	
13 – 17 years	11.55	-4.45	27.55	
Superficial				
Intercept	105.4			0.036
5 – 7 years	-2.31	-17.55	12.94	
8 – 12 years	13.46	-1.79	28.70	
13 – 17 years	1.51	-14.61	17.63	
**Sural nerve**				
Intercept	126.06			0.047
5 – 7 years	-6.85	-24.13	10.43	
8 – 12 years	11.21	-6.07	28.49	
13 – 17 years	-10.73	-29.13	7.67	
**Vagal Nerve**				
Intercept	130.79			0.013
5 – 7 years	-10.61	-32.77	11.55	
8 – 12 years	-3.28	-25.01	18.46	
13 – 17 years	4.56	-18.37	27.49	

β = β coefficient. CI = confidence interval.
